# Thoughts on air quality when the world is electrified

**DOI:** 10.1093/nsr/nwac145

**Published:** 2022-08-06

**Authors:** Ronald C Cohen

**Affiliations:** Departments of Chemistry and of Earth and Planetary Science, University of California, Berkeley, USA

We still live in a fossil-fuel-dominated world. Today, poor air quality is almost always associated with leaks of fuel into the atmosphere and unwanted byproducts of their combustion. However, a future where fossil fuels are replaced almost entirely with clean electricity is not far off. Preventing the worst possible outcomes of climate change demands that we create that world.

Along the path to that future, there will remain large sources of nitrogen oxides from combustion. The nitrogen oxides regulate the rate of chemistry of the atmosphere. The more nitrogen oxides, the faster things run and the closer to a source the most important chemistry occurs. Today, chemistry within cities is mostly local to those cities because NO_x_ is abundant. The other major ingredient in poor air quality are volatile organic molecules. In the future, when cars and industrial combustion are not dominant sources of volatile organic molecules, emissions from agricultural burning and wild fires, from our homes, non-combustion industrial uses, and from the biosphere will remain.

As we focus on this future, research is beginning to highlight the role for these sources. A common feature among them are emissions of monoterpenes. Monoterpenes (C_10_H_16_) are a class of molecule emitted directly by plants. Emissions directly from plants increase with temperature and have been proposed as a major contributor to poor air quality on the hottest days in Los Angeles [1,2]. They are also used in many household products and have been reported as part of a class of volatile chemical products (VCPs) [3]. In this issue, Wang *et al.* [4] highlight another anthropogenic source of monoterpenes that affects air quality: agricultural biomass burning.

Today, while we still have high NO_x_ in most cities, Wang *et al.* show (see their Fig. 4) that the atmospheric oxidation chemistry of monoterpenes accelerates formation of ozone. Monoterpenes are likely also becoming more important contributors to particulate matter, as suggested by Nussbaumer and Cohen [1]. Wang *et al.*’s point, that the chemistry changes at low NO_x_, is one indication of how we should approach thinking about an electrified future. Following their thinking, we should be keeping an eye on understanding the high NO_x_ chemistry that dominates in our cities today, but looking to understand the lower NO_x_ chemistry of cities in the future.

***Conflict of interest statement.*** None declared.
